# Skin Reactions and Quality of Life after X-Ray Therapy of Basal Cell Carcinoma

**DOI:** 10.1155/2012/825095

**Published:** 2012-12-20

**Authors:** Jette Skiveren, Maria Rudkjaer Mikkelsen, Helle Daugbjerg, Hans Christian Wulf

**Affiliations:** ^1^Department of Dermatology, Copenhagen University Hospital, Bispebjerg Hospital, Copenhagen 2400, Denmark; ^2^Research Unit of Clinical Nursing, Bispebjerg and Frederiksberg Hospital, Copenhagen 2400, Denmark

## Abstract

*Background*. Advanced basal cell carcinoma (BCC) is often treated by surgery or X-ray therapy. The consequences of X-ray therapy on the patients' health-related quality-of-life (HRQOL) have so far not been described. *Objectives*. To quantify quality of life in BCC patients before and after X-ray therapy compared with matched healthy controls. *Materials*. Twenty-five patients (mean age 69) with BCC completed the Dermatology Life Quality Index (DLQI) before and two weeks and three months after X-ray therapy and their results were compared with the DLQI scores for 25 matched controls. *Results*. Compared to the healthy controls the patients' DLQI score was significantly higher before and 2 weeks after X-ray therapy (*P* = 0.005; *P* = 0.000). The patients' DLQI score decreased significantly from baseline to three months after X-ray therapy (*P* = 0.024), when it became similar to that of the healthy controls (*P* = 0.819). Three months after X-ray therapy eight patients had no skin reactions, 11 had slight atrophy, pigmentation change, and/or some hair loss, four had patch atrophy, moderate telangiectasia, and/or total hair loss. *Conclusions*. BCC has a negative effect on patients' quality of life. The study shows that HRQOL normalises shortly after X-ray therapy, despite minor skin manifestations.

## 1. Introduction

Nonmelanoma skin cancers include basal cell carcinoma (BCC), squamous cell carcinoma, and more uncommon entities such as Merkel cell carcinoma [1, 2]. BCC can be treated with many modalities such as surgical excision, topical immunomodulations, Mohs micrographic surgery, photodynamic therapy, and X-ray therapy. X-ray therapy is an effective treatment and results in good function and cosmesis, why it could be considered as a first option in many cases such as high risk BCC; diameter > 2 cm, location on nose, lips, and ears, and rapid growth [2, 3]. X-ray therapy has been limited because of legal regulations and the high equipment and running cost of radiation devices, which discourage clinics from establishing the treatment [3]. 

Unlike many other medical conditions, skin disorders are visible and may result in recognition of illness and possible stigmatization. Approximately 80% of BCCs occur in the head and neck and may cause disfiguring skin changes with impact on the patient's body image and health-related quality-of-life (HRQOL) [4].

Two studies have used the Dermatology Life Quality Index (DLQI) to assess HRQOL in patients with BCC who have been treated with cryotherapy, curretage, excision, and Mohs for BCC surgery [5, 6], but no reports are available on HRQOL in patients with BCC treated by X-ray.

The aim of the present study was to quantify the HRQOL in patients treated by X-ray for BCC.

## 2. Material and Methods

The study population consisted of 25 patients with BCC treated by X-ray (40 Gy, KV 40 (median) in 10 fractions over two weeks) and 25 healthy controls without skin diseases matched by gender and age (Table 1). The controls without skin diseases were recruited among relatives to colleagues. The patients were enrolled consecutively for this descriptive study. 

To assess the specific impact of X-ray therapy, the validated short, self-administered questionnaire DLQI was used [5–7]. The DLQI consists of 10 questions relating to “the last week” answered on a scale from 0 (not at all), through 1 (a little), 2 (a lot), to 3 (very much), with a maximum possible score of 30. The higher the score, the more HRQOL is impaired [7–9]. The DLQI can be analysed under six headings, which include: (1) symptoms and feelings, (2) daily activities, (3) leisure, (4) work and school, (5) personal relations, and (6) treatment (Table 2). The questionnaires were completed immediately before X-ray therapy, and again two weeks and three months after treatment. A two-week follow-up was chosen because major skin reactions due to the X-ray are expected at this time, and the three-month follow-up was chosen because healing is expected to be completed at this time [3, 5, 6]. Data included demographic and clinical information. To evaluate the skin reactions three months after X-ray the RTOG/EORTC late morbidity scoring scheme was used [10].

The study protocol was approved by the Danish Data Protection Agency (CVR-nr. 11-88-37-29) and the Scientific Ethical Committee for Copenhagen County (KF 01 271885) and was conducted in accordance with the Declaration of Helsinki V. All participants gave written informed consent. 

### 2.1. Statistics

Data were analysed in SPSS Version 19.0 for Windows (SPSS, Inc., Chicago, IL, USA). The comparison of the patients with BCC and the healthy controls was performed using the Mann-Whitney *U*-test. The different effects of the X-ray therapy on DLQI at the different times were analysed using the Wilcoxon two-sample test. A two-sided *P* value <0.05 was considered statistically significant.

## 3. Results

Twenty-five patients with BCC (16 male, 9 female, mean age 70, range 44–95) and 25 healthy controls matched by gender and age participated in the study. Twenty-one (84%) of the BCCs were located on the head or neck, 3 (12%) on the trunk, and 1 (4%) on a leg. The lesion diameters at baseline were 7–60 mm (mean 25.9 mm) and had been present for a mean of 27 months (range 6–84 months) before X-ray treatment. The BCC diagnoses had in all cases been ascertained by a dermatologist (*n* = 25) and confirmed by a pathologist.

At baseline three (12%) patients had an ulcerative BCC. Eight (32%) patients had ulcerations immediately after the X-ray and 21 (84%) patients subsequently developed radiotherapy induced ulcerations, which had healed three months later. Three months after X-ray therapy nine patients had no skin reactions (grade 0, RTOG/EORTC late morbidity scoring scheme) [10], 12 had slight atrophy, pigmentation change, and/or some hair loss (grade 1), five had patch atrophy, moderate telangiectasia, and/or total hair loss (grade 2), none had marked atrophy and gross telangiectasia (grade 3), none had ulcerations (grade 4). Despite these changes we found a significant decrease in the overall DLQI scores from baseline to three months after X-ray therapy (*P* = 0.024) and from two weeks to three months after X-ray therapy (*P* = 0.002).

Scores in the subcategory “symptoms and feelings” significantly decreased from two weeks to three months after X-ray therapy (*P* = 0.004). Generally the scores in each of the subcategories and the overall scores of DLQI were very low (Table 2). The patients had a significantly higher DQLI score before and two weeks after X-ray therapy compared to the matched healthy controls (*P* = 0.007; *P* = 0.001) (Table 3). Three months after X-ray therapy the DLQI was similar for patients with BCC and healthy controls (*P* = 1.000).

## 4. Discussion

This study demonstrated a significant effect on patients' HRQOL from baseline to three months after therapy (Table 2).

**Figure 1:**
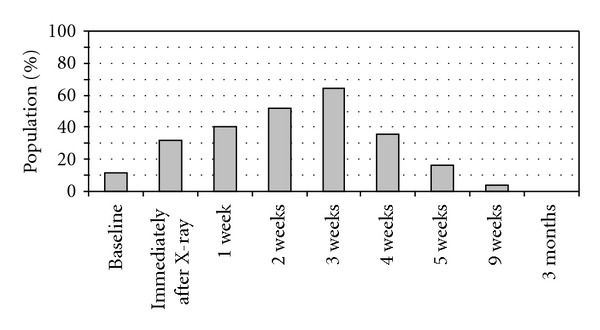
Percentages of patients with ulcerative BCC at baseline (immediately before X-ray therapy), and radiotherapy induced ulcerations at different time points after completed X-ray therapy (*n* = 25).

HRQOL is considered an important outcome in cancer research, and is associated with sequela such as depression, anxiety, and vulnerability [8]. Although BCC is not life-threatening, it may affect HRQOL, because BCC often results in skin changes and ulcerations after X-ray therapy and may result in recognition of illness (Figure 1) [5, 6]. 

In the present study the overall DLQI score was between 0.4–1.5 and this represents a very small effect on patient's HRQOL according to Finlay and Khan (Tables 2 and 3) [7]. The mean DLQI scores (maximum 30) in a normal population range from 0 to 0.5 [11], which was the case in our controls as well.

DLQI has been used in two other studies in relation to surgery for BCC (Table 4) [5, 6]. Both studies showed DLQI baseline 5.3 ± 4.1 and 2.4 ± 2.7, and a significant decrease in DLQI three months after surgery (1.3 ± 2.1 and 1.7 ± 2.9). The present study also showed a significant decrease in DLQI to normal level three months after treatment (from 1.2 + 1.5 to 0.4 ± 0.9) (Tables 2 and 3). X-ray therapy itself had no effect on HRQOL, which was less than for surgery (Table 4).

Within the first month X-ray therapy acute reactions are common, which may be reflected in DLQI's subcategory “symptoms and feelings”. Concern about the acute side effects of X-ray therapy seems not to be a problem after X-ray therapy [2], as the DLQI had the same levels as before X-ray and less than after surgery. 

The decline of the DLQI could be a result of confounders, but except for instructions in sun protection and care of ulcers, there was no difference between the healthy and the intervention group. Patients with BBC located at visible areas such as face or neck may suffer more than patients with BCC on at trunk and legs. This is confirmed by a separate analyse of face elements showing a significant difference in DLQI from before to 2 weeks after treatment, different from the total material.

A number of authors argue that DLQI may be too non-specific for this use [12–14]. The items seem not to capture such issues as scarring, disfigurement, and worry about recurrence or new lesions [12], which are frequent problems for patients with BCC. The questionnaire appears more tailored for chronic, benign skin conditions such as psoriasis or eczema, while it emphasizes physical complaints such as itchiness and irritation. The change in DLQI scores in this study could indicate the need for a more disease-specific instrument. The Facial Skin Cancer Index, a new and as yet less commonly used questionnaire, may prove useful, but there is a need for translation and validation for use in non-English speaking countries [4, 12, 15].

The present study shows that for 14 out of 25 patients more than a year passed before the patients sought medical advice, and the same tendency is seen in other studies [5, 16]. This could indicate that small lesions do not represent a major problem, which accords with what the DLQI scores indicate.

In conclusion, the diagnosis, BCC, has a negative effect on patients' HRQOL, and cure by X-ray therapy brought HRQOL levels back to those of healthy controls, despite minor skin manifestations.

## Figures and Tables

**Table 1:** Sociodemographic and medical characteristics of 25 Caucasian BCC patients and 25 healthy controls matched by gender and age. Duration between first notice and treatment time and diameter of lesions at baseline are stated.

Characteristics	BCC patients	Healthy controls
	Mean (range)	Mean (range)
Age (y)	69.5 (44–95)	69.5 (44–95)
Duration (months)	27.2 (6–84)	
Gender		
Male, *n* (%)	16 (64)	16 (64)
Female, *n* (%)	9 (36)	9 (36)
Location of BCC		
Head and neck, *n* (%)	21 (84)	
Trunk, *n* (%)	3 (12)	
Leg, *n* (%)	1 (4)	
Lesions diameter baseline, mm	25.9 (7–60)	

**Table 2:** Change in DLQI and subcategories over time among 25 BCC patients treated by X-ray.

Question	Baseline (A)	2 weeks after treatment (B)	3 months after treatment (C)	*P* value*		
				A-B	B-C	A-C
(1) Symptoms and feelings	0.7 ± 1.0	1.0 ± 0.8	0.3 ± 0.7	0.098	0.004*	0.093
(2) Daily activities	0.1 ± 0.3	0.1 ± 0.3	0.0 ± 0.0	1.000	0.157	0.157
(3) Leisure	0.2 ± 0.4	0.2 ± 0.6	0.0 ± 0.0	0.480	0.129	0.180
(4) Work and school	0.1 ± 0.2	0.0 ± 0.2	0.0 ± 0.0	1.000	0.317	0.317
(5) Personal relations	0.1 ± 0.3	0.0 ± 0.0	0.0 ± 0.0	0.157	1.000	0.157
(6) Treatment	0.1 ± 0.3	0.1 ± 0.3	0.0 ± 0.0	1.000	1.157	1.157

Total DLQI	1.20 ± 1.5	1.48 ± 1.2	0.36 ± 0.9	0.412	0.002*	0.024*

Mean score ±SD, Wilcoxon Signed Rank Test, sig. (2-tailed), *Significant.

**Table 3:** Total DLQI scores for patients with 25 BCC baseline and after X-ray therapy and 25 healthy controls matched by gender and age.

	Healthy controls	Patients with BCC	*P* value patients versus healthy controls
Baseline	0.3 ± 0.5	1.2 ± 1.5	0.005*
2 weeks after X-ray		1.5 ± 1.2	0.000*
3 months after X-ray		0.4 ± 0.9	0.819

Mean score ± SD, Mann-Whitney *U*-test. (2-tailed), *Significant.

**Table 4:** Changes in total DLQI scores between baseline and after different treatments. Mean score ±SD.

	Treatment	Age ± SD (range)	Duration, months (range)	Baseline	1-2 weeks after treatment	3 months after treatment
This paper	X-ray	69.5 ± 11.5 (44 − 95)	27.2 (2 − 84)	1.2 ± 1.5 (*n* = 25)	1.48 ± 1.2 (*n* = 25)	0.36 ± 0.9 (*n* = 25)
Blackford et al. [5]	Surgery	65 (35 − 81)	25 (1 − 240)	5.3 ± 4.1 (*n* = 44)	8.7 ± 9.6 (*n* = 34)	1.3 ± 2.1 (*n* = 37)
Rhee et al. [6]	Surgery	62.2 ± 15.4		2.4 ± 2.7 (*n* = 121)		1.7 ± 2.9 (*n* = 101)
